# Overview of Hepatocellular Adenoma in Japan

**DOI:** 10.1155/2012/648131

**Published:** 2012-09-02

**Authors:** Motoko Sasaki, Yasuni Nakanuma

**Affiliations:** Department of Human Pathology, Kanazawa University Graduate School of Medicine, Kanazawa 920-8640, Japan

## Abstract

Hepatocellular adenoma (HCA) is generally a benign hepatocellular tumor arising in a nonfibrotic/cirrhotic liver, and recently four major subgroups were identified based on genotype and phenotype classification from Europe. HCA is rare in Asian countries including Japan, and there have been few studies regarding the subgroups of HCA in Japan. We surveyed subgroups of HCA in 13 patients (7 women) in Japan, based on the phenotypic classification. As results, we identified 2 hepatocyte nuclear factor (HNF) 1**α**-inactivated HCAs (15%), two **β**-catenin-activated HCAs (15%), 5 inflammatory HCAs (39%), and 4 unclassified HCAs (29%). The use of oral contraceptives was found only in 2 unclassified HCAs (29%). Rather low percentage of female patients and use of oral contraceptives appear to be common clinicopathological features in Japan and also East Asian countries. Furthermore, a group of possible inflammatory HCAs characterized by strong immunoreactivity for serum amyloid A (SAA) was found in patients with alcoholic cirrhosis. The inflammatory HCA/SAA-positive hepatocellular neoplasm in alcoholic cirrhosis may be a new entity of HCA, which may have potential of malignant transformation. Further studies are needed to clarify genetic changes, monoclonality, and pathogenesis of this new type of hepatocellular neoplasm.

## 1. Introduction

Hepatocellular adenoma (HCA) is benign monoclonal tumors occurring essentially in young women taking oral contraceptives (OCs) [1]. Eighty-five percent of cases occur in young women; HCA is rare in children, men, and the elderly [1–5]. The incidence of HCA is about 3-4 per 100,000 populations in Europe and North America [1], but lower in Asian countries, including Japan. HCA represents a heterogeneous entity, recently subclassified into several groups according to genotype and phenotype [2–6]. Recurrent mutations were identified in the HNF1A (hepatocyte nuclear factor 1 A) gene encoding the HNF1*α*, in the CTNNB1 (catenin (cadherin-associated protein), *β*1) gene coding for *β*-catenin, and recently in the interleukin 6-signal transducer gene encoding the signaling coreceptor gp130 [7]. In inflammatory HCA, about 60% of patients have small in-frame deletions in gp130 [7] and additional 12% carried activating STAT3 mutations [8]. Genotyping allowed the identification of three subtypes: HNF1*α*-inactivated HCA (35%–50% of cases), *β*-catenin-activated HCA (15%–18% of cases), and inflammatory HCA (40%–55% of cases), which could be identified by immunohistochemistry on paraffin-embedded materials [2–6]. Less than 10% of HCAs did not express any of these phenotypic markers (unclassified HCA). Few reports have examined the clinicopathological features and subtypes of HCA in Asian countries, including Japan [9–11]. Therefore, we have started to survey patients with HCAs in Japan and compared clinicopathological features with previous reports from other area.

## 2. Phenotypic Classification of HCA Subgroups

Immunohistochemical markers used for the phenotypic classification include liver fatty acid binding protein (LFABP), glutamine synthetase (GS), *β*-catenin, and serum amyloid A (SAA). LFABP-negative, GS-positive and/or nuclear *β*-catenin-positive, and SAA- positive HCAs are regarded as HNF1*α*-inactivated, *β*-catenin-activated, and inflammatory HCAs, respectively [2–6]. The remaining HCAs without specific markers are regarded as unclassified HCAs.

## 3. Clinical and Pathological Features of HCAs in Japan

Table 1 summarizes the clinical and pathological features of each subtype based on the new phenotypic classification [10]. We retrieved 13 HCA cases from our pathological files from 1997 to 2011 and surveyed a phenotypic classification of HCAs. In addition, we found 7 patients with alcoholic cirrhosis and hepatocellular neoplasms showing the immunoreactivity for SAA as same as inflammatory HCAs in same series. Since HCA usually arises in nonfibrotic/cirrhotic livers, “HCA in cirrhotic liver” appears to conflict with a general concept. Therefore, we tentatively called these possible HCAs as “SAA-positive hepatocellular neoplasms” [12] and did not include them in the present study. We will discuss on this possibly new type of HCA, as described below.

**Figure 1:**
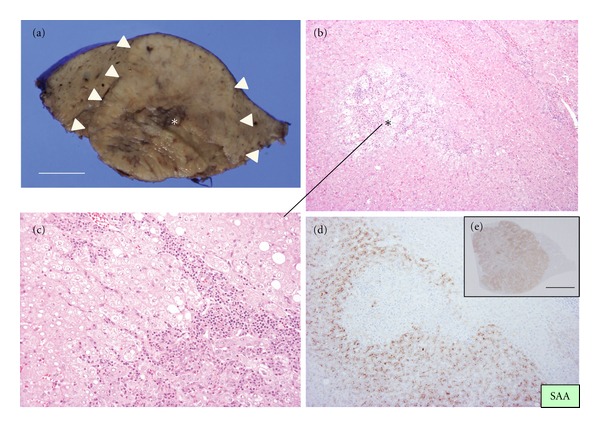
Inflammatory hepatocellular adenoma (HCA). (a) Cut surface shows whitish nodular lesion sized 3 cm in diameter (arrowheads). There was no capsule and the border between nodule and background liver is unclear. Congestion or bleeding is observed inside the nodule (asterisk). Bar = 1 cm. (b) Border of HCA and background liver. Ductular reaction is focally seen in HCA (asterisk). HE, ×100. (c) Ductular reaction with lymphoid cells infiltration in HCA. HE, ×200. (d) Strong expression of serum amyloid A component (SAA) in HCA in contrast to the background liver. Immunostaining for SAA and hematoxylin. ×100. (e) bar = 1 cm.

Patients with HCAs included 6 men and 7 women and their age ranged 15–68 yrs (41.0 ± 13.9 yrs). We identified 2 HNF1*α*-inactivated HCAs (2 women), two *β*-catenin-activated HCAs (one woman), 5 inflammatory HCAs (one woman), and 4 unclassified HCAs (3 women). The use of OCs was found in only 2 unclassified HCAs (29% of women).  Figure 1 shows the representative histology and immunohistochemical findings of inflammatory HCAs. Two inflammatory HCA patients (one woman) were obese (BMI >25) and 2 inflammatory HCAs (all men) had diabetes. Mild hepatic fibrosis liver was seen in the background in 3 inflammatory HCAs (60%) and 2 unclassified HCAs (50%). Steatosis was seen in the background liver in one *β*-catenin-activated HCA, 4 inflammatory HCAs (36%), and 2 unclassified HCAs (50%). Six patients (46%) had multiple HCAs; one HNF1*α*-inactivated HCA, 2 inflammatory HCA, and 3 unclassified HCAs. All multiple HCAs were of the same type in each case. The tumor was significantly larger in *β*-catenin-activated HCAs than in other subtypes. The association of hepatocellular carcinoma was seen in only one case of unclassified HCA. A definite diagnosis of HCC was made based on cytological abnormalities, a loss of reticulin fiber in a focal area in this HCA. In addition, this area showed the immunoreactivity for glypican-3, which is a marker of HCC.

## 4. Features of HCAs in Japan: A Comparison with Previous Reports

Table 2 summarizes a comparison of HCAs reported from Japan [9, 10], Europe [3], and China [11]. Our study revealed different clinical and pathological features of HCAs in Japan from those in Europe and North America [1–5, 9, 10]. The features of HCAs in Japan can be listed as follows: (1) a half of HCAs occur in men; (2) a use of OCs is infrequent; (3) a rate of HNF1*α*-inactivated HCAs is rather low.

**Gender and OCs**

Compared with previous studies reported from Western countries [1–5, 13], in which more than 90% of patients with HCAs were women, lower percentage of female patients appears to be a feature of HCAs in Japan [9, 10]. A half of HCAs occur in men in our study. This result agrees with a previous study from Japan, in which about 60% of patients with HCAs were women [9]. Interestingly, the percentage of female patients was further less in the study from China [11]. The use of OCs is infrequent in HCAs in Japan in our study and a previous report [9], whereas the use of OCs was noted in about 80–90% of female patients with HCAs in Europe [3, 13]. The use of OC was also infrequent in China [9]. A lower rate of obesity might be a reason for the difference. The high frequency of men seems to be related to rather low rate of HNF1*α*-inactivated type. In addition, HCC, not HCA, is generally suspected for hepatic nodules arising in men. In fact, partial hepatectomy was performed in male patients in our survey, since HCC was suspected. Same nodules might be diagnosed in HCA in women and would be followed up without resection. The previous study in Japan reported a higher rate of male patients with HCAs associated with predisposing factors, such as glycogen storage disease and anabolic hormone treatment for aplastic anemia [9]. Such predisposing factors were not noted in the patients with HCAs in our recent study [10]. Taken together, lower percentage of female patients and use of OCs may be common characteristics of patients with HCAs in East Asia.

**Tumor Size and Multiplicity**

Tumor size ranged 1–31 cm and mean of tumor size was 5.3 cm, 11.3 cm, 8.3 cm, and 7 cm in each study (Table 2) [3, 9–11]. A mean tumor size is rather big in the previous study from Japan reported in 1995 [9]. Small HCAs may be able to be detected earlier because of recent progress of imaging modalities. Multiple tumors were detected in 46% and 39% of patients in the present study and study from France [3], respectively. In contrast, only 6% was diagnosed as multiple lesions in the study from China [11]. The reason for this difference is unclear.

**Association of HCC**

The malignant transformation of HCA to HCC has been described in previous studies [2, 3, 14–16]. Association of HCC is 8% in our present study and it ranged 2–6% in other studies [3, 9, 11]. The risk of HCC is linked to *β*-catenin mutation [2, 3, 14]. Larger size and male gender are also related to the association of HCC [11, 14]. The patient with HCA with malignant in our present study was a man having unclassified HCA, 5 cm in size. In addition, coexistence of chronic hepatitis B virus infection was suggested to be a risk factor for malignant transformation in the report from China [11].

**Subgroups**

We have reported for the first time a proportion of subgroups of HCAs in Japan and Asian countries, to our knowledge [10]. In our present study, a percentage of HNF1*α*-inactivated HCA was lower than the study from France [2, 3]. Recently, Fukushima et al. surveyed subgroups of 14 Japanese patients with HCAs and reported the proportion was as follows: HNF1*α*-inactivated, 18%; HNF1*α*-inactivated and *β*-catenin—activated 18%, *β*-catenin—activated, 12%; inflammatory HCA, 29%; unclassified, 24% [17]. A lower rate of HNF1*α*-inactivated HCA may be a feature of HCAs in Japan. Since HCA associated with mutant HNF1A, a major type of HNF1*α*-inactivated HCA, occurs almost exclusively in women [2–6], higher rate of male patients may be a reason for a lower rate in this subgroup. Inflammatory HCA is a major subgroup in this study, consistent with recent studies in Europe [3, 13]. Therefore, it is conceivable that inflammatory HCA may be a common major subgroup of HCAs, irrespective of race and region. It is not clear why the rate of unclassified HCA was rather high in our survey. These unclassified HCAs did not show the immunoreactivity for CRP, another marker of inflammatory HCA. Furthermore, these unclassified HCA did not show histological characteristics of inflammatory HCA, such as ductular reaction and inflammatory cell infiltration. Mild steatosis was seen in one unclassified HCA.

## 5. Serum Amyloid A-(SAA) Positive Hepatocellular Neoplasm in Alcoholic Cirrhosis: A Possible New Subtype of HCA

**Figure 2:**
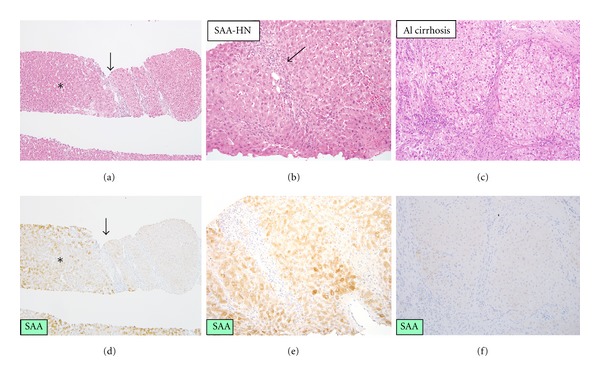
Serum amyloid A-positive hepatocellular neoplasm associated with alcoholic cirrhosis. (a) The boundary between serum amyloid A-positive hepatocellular neoplasm (SAA-HN) (asterisk) and nontumor tissues. Arrow indicates the boundary. ×40. (d) Serum amyloid A is expressed in the tumor, whereas it is negative in the background liver. Immunostaining for serum amyloid A, ×40. (b) Serum amyloid A-positive hepatocellular neoplasm (SAA-HN). Inflammatory cells infiltration, focal sinusoidal dilatation and ductular reaction are seen in the tumor. HE, ×200. (e) The cells in the nodule show extensive granular immunoreactivity for serum amyloid A. Immunostaining for serum amyloid A, ×200. (c) Background liver shows established micronodular cirrhosis consistent with alcoholic cirrhosis. HE, ×200. (f) The background liver shows negative immunoreactivity for serum amyloid A. Immunostaining for serum amyloid A, ×200.

Our previous study highlights a group of hepatocellular nodules in alcoholic cirrhosis which conflicts with the general concept of “HCAs” [10]. In fact, the liver lesion with solid mass arising in fibrotic/cirrhotic background is not supposed to be an HCA according to the diagnostic algorithm proposed in WHO classification 2010 [2]. Histologically, these nodules showed features of inflammatory HCAs, such as sinusoidal dilatation, inflammatory cell infiltration, and ductular reaction (Figure 2). Immunohistochemically, these nodules showed strong immunoreactivity for SAA as same as inflammatory HCAs (Figure 2). Immunostaining for GS showed weak and diffuse pattern in these nodules, which was different from focal nodular hyperplasia (FNH) showing map-like pattern. Taken together, we named this unique type of possible HCA as “SAA-positive hepatocellular neoplasm” [12] and have been doing further studies. SAA-positive hepatocellular neoplasms included some hypervascular hepatic nodules showing similar imaging findings to HCC; the so-called FNH-like nodules, or drinker's nodules, occur in severe alcoholic fibrosis or cirrhosis [18–20]. In our survey, six patients with SAA-positive hepatocellular neoplasms were found in 10 patients with the so-called FNH-like nodules and alcoholic cirrhosis. Further studies including molecular analysis are necessary to establish this new type of nodular lesions.

It is well known that alcohol is a risk factor of HCC. In addition, metabolic syndrome is a newly identified risk factor in chronic liver disease and HCC [21–23]. Alcohol, obesity, and fatty liver diseases are frequent in inflammatory HCAs [2, 3]. So, HCAs may be precursor lesions of HCC in metabolic syndrome. Similarly, SAA-positive hepatocellular neoplasms may be precursor lesions of HCC. SAA-positive hepatocellular neoplasms showed different features from HCC in the immunoreactivity for GS and glypican-3 [12]. Most SAA-positive hepatocellular neoplasms were negative for glypican-3 in our recent study [12]. Therefore, glypican-3 is useful to differentiate SAA-positive hepatocellular neoplasms from HCCs. HSP70 is also reportedly useful to differentiate benign and malignant tumors [24], so this marker may be useful. The CD34-positive capillarization of sinusoid was seen in both SAA-positive hepatocellular neoplasms and HCCs in our preliminary study, so CD34 may not be a definite marker for differential diagnosis. However, it should be noted that histological diagnosis between HCA and well-differentiated HCC may be difficult in some cases. Interestingly, Farges et al. have recently reported changing trends in malignant transformation of HCA [14]. Prevalence of malignancy within HCA is 10 times more frequent in men than in women in their study [14], and they propose that management of HCA should primarily be based on gender. Whereas oral contraception is a classical cause of HCA in women but a marginal cause of HCC, the metabolic syndrome appears as an emerging condition associated with malignant transformation of HCA in men and is the likely predisposing condition for HCC in this setting [14]. Taken together, clinical and pathological features SAA-positive hepatocellular neoplasm, a possible inflammatory HCA, should be examined in larger series to confirm the significance of unique lesion.

## 6. Concluding Remarks

Clinical and pathological features of HCAs appear to be different in East Asia including Japan and Western countries. Lower percentage of female patients and use of OCs may be common characteristics of HCAs in East Asia including Japan. Our recent studies highlight a characteristic group of hepatocellular neoplasms arising in alcoholic cirrhosis, which share features with inflammatory HCAs. These SAA-positive hepatocellular neoplasms may be a new type of hepatocellular tumors having a potential of malignant transformation in alcoholic patients. 

## Figures and Tables

**Table 1:** Clinical and pathological findings of hepatocellular adenoma.

	HNF1*α*-inactivated (*n* = 2)	*β*-catenin-activated (*n* = 2)	Inflammatory HCA (*n* = 5)	Unclassified (*n* = 4)
Patients				
Age; mean ± SD (range)	40.5 ± 4.9 (37–44)	28.5 ± 3.5 (26–31)	40.2 ± 22.8 (15–68)	33.5 ± 14.1 (16–45)
Sex (M/F)	0/2	1/1	4/1	1/3
Oral contraceptives	0	0	0	2
Alcohol >40 g/day	0	0	2	0
BMI >25	0	0	2	0
Other clinical background	Portal hypoplasia 1	Hyperlipidemia 1	Diabetes 2	FAP 1
Background liver				
Steatosis	0	1	3	2
Fibrosis (F1/2/3/4)	0	0	3 (2/1/0/0)	2 (1/1/0/0)
Tumor				
Tumor size; mean ± SD (range)	4.0 ± 1.4 (3–5 cm)	17.5 ± 2.1* (16–19 cm)	3.9 ± 2.6 (1.6–9 cm)	4.4 ± 2.6 (1.5–7 cm)
Unique/multiple	1/1	2/0	3/2	1/3
Steatosis	1	1	2	1
Association of HCC	0	0	0	1

HNF1*α*: hepatocyte nuclear factor 1*α*; HCA: hepatocellular adenoma; M: male; F: female; BMI: body mass index; FAP: familial adenomatous polyposis; HCC: hepatocellular carcinoma; *P < 0.05, compared to other groups.

**Table 2:** Hepatocellular adenoma in Japan: a comparison with previous reports.

	Sasaki et al., 2011 [10]*	Konishi et al., 1995 [9]	Lin et al., 2011 [11]	Bioulac-Sage et al., 2009 [3]
	(*n* = 13)	(*n* = 58)	(*n* = 191)	(*n* = 128)
Age; mean (range)	41 (15–68)	34	39 (5–79)	41 (21–66)
Female	54%	62%	38%	91%
Oral contraceptives	29%	8%	4.2%	78%
Alcohol >40 g/day	15%	nd	nd	12%
BMI >25	15%	nd	nd	33%
Tumor size; mean (range) (cm)	5.3 (1.5–19)	11.3	8.3 (2–31)	7 (1–18)
Multiple	46%	16%	6%	39%
Association of HCC	8%	2%	6%	4%

	HNF1*α*-inactivated: 15%			HNF1*α*-inactivated: 38%
Subtypes	*β*-catenin-activated: 15	nd	nd	*β*-catenin-activated: 6%
	IHCA: 39%			IHCA: 52%
	Unclassified: 31%			Unclassified: 5%

HNF1*α*: hepatocyte nuclear factor 1*α*; IHCA: inflammatory hepatocellular adenoma; BMI: body mass index; *with modification.
